# The Practical Utilization of Ozone in the Treatment of Disease*Read before the Buffalo Medical Association, June 12, 1883.

**Published:** 1883-08

**Authors:** F. W. Bartlett


### BUFFALO

### Medical and Surgical Journal.

VOL. XXIII. AUGUST, 1883. No. 1.

### Original Communications.

### THE PRACTICAL UTILIZATION OF OZONE IN THE TREATMENT OF DISEASE.*

BY F. W. BARTLETT, M. D.

Mr. President, and Members of the Association—While it would be interesting to review, fully, the history of ozone and give due credit to all illustrious scientists who have contributed to our knowledge of it, time will only permit me to make a very brief summary of what we may be fairly said to know of this mysterious component of the atmosphere. Referred to by Homer in the Odyssey and Iliad as the "Odor of thunderbolts," it has been repeatedly described as a sulphurous vapor, noticeable in thunder storms and certain conditions of the atmosphere.

Dr. Cornelius B. Fox, of London, England, has summarized these observations in the opening chapter of his admirable work upon ozone, and from it I shall largely quote.

The first observation recorded of the peculiar smell of ozone artificially produced, was by Van Marum, of Holland, about 1785, who, having passed electric sparks through oxygen, discovered by Priestly in 1774, observed the odor we call ozone,

* Read before the Buffalo Medical Association, June 12, 1883.

and also that the gas thus produced oxidized mercury. Cavallo, early in this century, noticed that electrified air had a purifying effect upon animal and vegetable matter when decomposing, and employed it as a disinfecting wash in fetid ulcers.

In 1826 Dr. John Davy recognized it in the atmosphere and published a formula for the preparation of chemical tests for its detection.

In 1839, Christian Frederick Schonbein, Professor of Chemistry at Basle, and the inventor of gun-cotton, while engaged in some experiments on the decomposition of water by voltaic electricity, was astonished at the development of a peculiar odor similar to that emitted during discharges from the electrical machine. He showed that it was due to the existence of a body possessing, like oxygen, the property of most readily entering into chemical combinations. His announcement of this, and subsequent communications, drew the attention of the scientific world to the remarkable properties of this rediscovered body which he named ozone, from a Greek word signifying "to emit an odor."

During the forty years which have since elapsed, most of the scientists of the century have studied ozone, and I only refrain from giving their names and special discoveries from lack of space. Over and above them all, by common consent, towers the name of Schonbein, whose preliminary discoveries invited attention to its study, and whose zeal never wavered till his grand genius passed on to that illimitable existence where all mysteries are explained.

Ozone has been named "electricized oxygen," "allotropic oxygen," "nascent oxygen," "active oxygen," excited oxygen."

Omitting much that has been claimed and thereby neglecting deserved honors to many eminent explorers, it must suffice to say that the present accepted theory of the nature of ozone is that it is oxygen in a highly electrified state and possessing oxidizing qualities not found in ordinary oxygen. The formula of oxygen being $O_2$, Dr. Odling thinks that the molecule of ozone contains three atoms of oxygen and that its formation means the condensation of oxygen into two-thirds its former volume. It may be described as oxygen *plus* force. It is never obtained in a pure form, but always mixed with a certain proportion of oxygen. Ozone reverts slightly to ordinary oxygen at 100° Fahrenheit and wholly at 237° F. It is produced by various forms of electric apparatus, among which may be mentioned those of Siemens, Beanes, Ladd, and Houzeau; by the electrolysis of water, by phosphorus partially immersed in water, by the action of strong sulphuric acid upon potassium permanganate, by dispersing water in a pulverized form through the air, by the introduction of a hot glass rod into a vessel of air through which the vapor of ether has been diffused, and by the slow oxidation from exposure to light of sulphuric ether, chloroform, oil of turpentine, lemons, linseed, cinnamon, bergamot and most essential oils. The turpentine sold in the shops often contains fifty per cent. of its volume of one of these oxidizing principles. Whenever a chemical reaction takes place in the air ozone is said to be produced. It is insoluble in solutions of the acids and alkalies, alcohol, ether, essential oils and water. It is stated by Andrews to be three and one-half times heavier than oxygen. It acts upon most substances as an oxidizing agent of great power, converting indigo into isatine, the black into the white sulphate of lead, the yellow ferro-cyanide into the red cyanide of potassium; arsenic, antimony, iron, manganese, zinc, tin, lead, bismuth, silver, nickel and mercury, are all oxidized by it. It also transforms many of the lower oxides into peroxides. Schonbein states that nitrites may be changed into nitrates by ozone *only*.

Its corrosive powers and property of destroying most organic substances are remarkable; concentrated, it excels chlorine itself. Wood, straw, cork, starch, vegetable colors, rubber, pure and galvanized, fats, alcohol and albumen, are oxidized by this agent. It is said to be absorbed by the blood corpuscles with great rapidity. It possesses the quality of oxidizing putrid exhalations. In illustration, the following experiment performed by Drs. Wood and Richardson is given in Dr. Fox's work: "In 1854, a pint of the blood of an ox, coagulated, was exposed to the air until it was quite putrid and the clot was softening. At the close of the year, the clot having re-dissolved, as a result of alkaline decomposition, the blood was a most offensive fluid. In 1862, the fluid was found to be so offensive as to produce nausea when the gases evolved from it were inhaled. Drs. Wood and Richardson subjected it to a current of ozone from Siemens' apparatus; gradually the offensive smell passed away and the fluid blood became quite sweet. The dead blood, moreover, coagulated as the products of decomposition were removed, and so perfectly, that a new clot exuded serum."

Into the consideration of antozone, an element said to be always present when ozone is formed, I do not propose, for want of time, to enter. It affords, of itself, a curious study, and perhaps may be properly considered a peroxide of hydrogen.

We come now to the important question, does the atmosphere contain ozone? In 1865, the French Academy of Sciences appointed a committee to ascertain whether it existed in the atmosphere, if any tests would reliably prove its existence, or could be discovered. This committee differed in their conclusions, but the experiments of Schonbein, Houzeau, Andrews and Fox, seem to settle the question in the affirmative.

At the Agricultural College, Lansing, Michigan, the following test is employed: Ten parts starch, two hundred parts water; heating till the starch gelatinizes, and then dissolving in this one part of pure iodide of potassium. This paste is then spread evenly with a flat brush on sheets of paper free from sizing, which are rapidly dried by stove heat, without exposure to sunlight, and then stored up in a covered jar and kept from exposure to light. The starch, water and iodide of potassium must be pure. This paper, moistened, is exposed to the air, and the amount of ozone present is estimated as it changes from white to dark brown.

Ozone, liquefied under pressure, has an intense blue color. It is more abundant in winter and spring than in summer and autumn, probably, from less organic decomposition. Statements have been made as to the power of ozone upon germs, but, upon this point, Dr. Fell will enlighten you.

"It is said cloth goods will not dye when ozone is absent from the air, and that the effect of thunder storms in increasing the power of the mordant is remarkable." Dust storms increase coloration of tests but slightly.

Ozone is more abundant in days of rain, also of snow, with southern winds than northern, and from the sea. Dr. Witherill noted abundance of ozone in the public grounds at Washington at night, but none in the streets. At Amiens Cathedral, the higher the tests were hung the greater the discoloration. It is always absent from the air of inhabited rooms, hospitals, prisons, etc. The growth and vigor of plants has been said by Mr. Carey Lea to be affected by ozone, and I find that in sick rooms, where ozone is used, plants almost invariably shed their leaves. I have attributed this to the destruction of the carbonic acid, so important to plant respiration, by the ozone. Plants furnish ozone by daylight, but not at night.

Dr. Kedzie, Prof. of Chemistry, Agricultural College, Lansing, Mich., says: "Ozone oxidizes all the metals except gold and the platinum group. It instantly burns phosphuretted hydrogen; oxidizes hydrochloric acid so that it will dissolve gold leaf; oxidizes iodide of potassium, setting free the iodide and forming caustic potash; sets free bromine; will also oxidize the deadly carbonic oxide into the comparatively innocent carbon dioxide or carbonic acid. Carbonic oxide is one of the most deadly gases known. It gives no warning of its presence by its odor. The power of ozone to oxidize this deadly poison is a fact of prime importance. Ozone will instantly destroy the horrible smell of rotten eggs. Schonbein found that air, made foul by exposure one minute to four ounces of highly putrid meat, was disinfected by an equal quantity of air containing one part ozone in 3,240,000 parts of air."

Ozone has an irritating effect upon the mucous surfaces of the throat, when highly concentrated, and has been thought, when in excess in the atmosphere, to produce influenza.

The reports by various physicians upon the supposed effect of ozone in the atmosphere, in the causation of epidemics, are too loose and contradictory to establish anything in this direction. The evidence, however, is in favor of an atmosphere with high ozone readings. Dr. Ireland has made many experiments on animals with ozonized air. He states that ozonized air accelerates the respiration, and, we may infer, the circulation.

Ozonized air excites the nervous system. Animals may be subjected to a considerable proportion of ozone without serious injury, but, in the end, ozone produces effects which may continue after its withdrawal and cause them to die. Dr. Swartzenburg has arrived at a similar conclusion. He found that a rabbit made to breathe air mixed with only one-two-thousandths of its weight of ozone would die in two hours. Pigeons exhibit a greater tolerance, while frogs resist it completely, so long as they are supplied with water. An air so charged with ozone would be an *irrespirable* air and would affect with varying intensity all who breathed it. The odor of ozone is so powerful that one-millionth of it in the air is said to have a decided smell of the gas. The air of the country is said by Houzeau, to contain not more than one-four-hundred-and-fifty-thousandth of its weight or one-seven-hundred-thousandth of its volume. The density of ozone is one-six-hundred-and-eighty-five-one-thousandth. The conclusions regarding ozone, says Dr. Fox, may be confidently summarized as follows:

"1st. A deficiency of ozone in the air in all probability predisposes to disease, particularly in the epidemic form, by virtue of the depressing and debilitating effect of such air in consequence of its feeble power of oxidizing animal debris.

"2d. A permanent diminution in the normal amount of active oxygen favors the development of chronic diseases characterized by mal-nutrition, imperfect oxidation and degeneration of tissues." Prof. Polli has shown that a dove will live longer in an atmosphere composed solely of ozone than in confined air which is vitiated by its own emanations.

Ozone abounds on the windward, and is deficient on the leeward side of cities, and is probably used up in the destruction of organic matters.

I omit description of various ozonoscopes and test papers for estimating ozone in the atmosphere as not within the intent of this paper.

Thus much of the history of ozone, the opinions of physicians and scientists upon its nature, its existence in the atmosphere in varying degree and the means used for its detection. So far we have no mention of its utilization, as artificially produced, in the treatment of zymotic disease, nor can I find, by diligent inquiry, that it has been systematically employed for this purpose. Compelled, then, to submit my own experience and to claim for it originality, I hope not to offend by egotistical pretensions or to be improperly influenced by enthusiasm.

In the month of October, 1876, I had the honor to read a paper before this association with similar caption to that now used. In it I detailed my experiments with ozone, artifically produced, with reports of forty cases treated in air thus modified. I claimed remarkably successful results in cases of peculiar severity and invited the association to use the crude apparatus exhibited. I felt then that my ideas would seem utopian and that I should be left to prosecute, individually, further trial of ozone. Nearly seven years have since passed away, and with the added results of 2,000 cases of disease treated in ozonized air, I again bring the subject before you. These cases are *essentially* zymotic, comprising scarlet fever, diphtheria, whooping-cough, typhoid and malarial fever, phthisis, hay fever, measles, dysentery, influenza, pneumonia, etc. All have been treated by simple exposure of the patients to air more or less ozonized and persistently or intermittingly employed.

In diphtheria the mortality has been five per cent.; in scarlet fever four per cent.; in typhoid fever ten per cent.; in whooping-cough, mortality none ; measles, none ; hay fever, none ; phthisis, relief ; dysentery, none. It is only in general terms that I make this statement. To be more specific I will cite illustrative cases: The first case treated by me in ozonized air was a child of Mr. G. M., Butler street, in 1876, born with a bright scarlatina rash. I immediately placed it in a strongly ozonized air, with no expectation of a favorable result, as such cases invariably die. The child, however, kept up a diaphragmatic jerk for thirty-six hours, when regular respiration was established, and it recovered with the usual desquamation and no sequelæ. An older child had been under treatment for two weeks, in ordinary atmosphere, and had a tedious convalescence, with cervical swellings and otitis. No medicine was given the case first cited.

Two weeks later, I was called to attend a child, aged three years, of Mr. C., Georgia street, for diphtheria, a truly malignant case. Had been ill three days ; fauces covered, and posterior and anterior nares filled by the exudate ; face purple ; deglutition impossible, and symptoms of impending convulsions. Predicted speedily fatal termination, but proposed, for the possible benefit of four other children compelled to occupy the same apartment, the use of ozone. Consent given, a generator was placed close by the head of the child, and proto-iodide hydrarg. given in one-fourth grain doses every three hours. Convulsions occurred every three or four hours for four days, the child being in a comatose condition in the interim. At the end of that time consciousness was partially restored, convulsive action ceased, the exudate was sufficiently loosened to be removed from the nostril, and the child recovered. The other members of the family, six in number, remained exempt from the disease. As further illustration of the apparent value of ozone in limiting the spread of scarlatina, the following cases may be cited : Mrs. B., residing on Peach street, had a child aged seven years taken, in November, 1879, with scarlatina maligna. Six other children were simultaneously exposed. The residence was an ordinary small one-story cottage, containing five rooms, two being bed-rooms. In one of these the child was placed with special ozone generation, and a generator was also placed in the dining-room adjoining the bed-room. The children were confined to the front room, except at meal time, when, the door of the sick room being closed, they took their meals in the dining-room. With these precautions, all escaped the disease, and the patient made a good recovery. Mrs. H., residing on Oak street near Eagle, had child taken with scarlet fever in the summer of 1879. Directed isolation in upper front room of two-story house, mother to nurse, and no one else to be admitted to the room. Ozone used; child recovered; no other cases. Two years later, disease appeared in same family (medical attendant not known to me) and three patients, about two, eleven and eighteen years of age, died. I can go on for hours with reports of cases successfully treated in ozonized air, but time will not permit. Rather let me specify the general effects apparently following its use:

1st. Upon the throat in diphtheria: and here let me say, that I rarely modify a diphtheritic exudate by topical applications, except by insufflation of pulverized chloride of sodium as recommended by me in a paper read before the association, I think in 1872. Lately, I add to this quinine and sulphur. Usually, I do nothing more locally, except to subject the throat to the action of ozone. Liquefaction takes place promptly and fetor is almost always entirely suppressed. The exudate usually disappears—in mild cases always—by the third day, and, as a rule, there is no secondary return. The general treatment is not novel and need not be described.

In scarlet fever the object is to change the atmosphere as fully as possible, but especially locally, as the general cutaneous surface affords an outlet to the poison. In typhoid fever, general ozonization, isolation, and purification of water and fluids ingested. Whooping-cough is treated by ozonization and the effects are in many cases truly surprising. I cite a special case: A child of a family residing on Tupper street, near Virginia, was brought to my office by the mother, and while there had a paroxysm of such severity that I thought respiration must cease. Expostulating with the mother for exposing the child, she said she expected death at any spasm and dare not leave the house without the child. Cough had existed for about three weeks. Was placed that night in ozonized air and had no paroxysm for twenty-four hours, and convalesced, with two other children similarly affected, very rapidly.

In hay fever results are usually, but not invariably, favorable; in asthma, doubtful; in dysentery, valuable by the favorable disinfection and deodorizing of the apartment; in croup, membranous and diphtheric, no certain advantage. In three cases of puerperal fever, where a generator was placed within the covering of the bed, there was prompt removal of foul odors and the patients recovered, but whether this was due to the local disinfection, may be doubted.

I have introduced ozone into the treatment of eight cases of phthisis pulmonalis, all in advanced stages, without expectation of cure, but in all there was lessening of cough, perspiration, diarrhœa, and in no one of them was there local or general œdema before dissolution. They all professed to be relieved by it and asked for its continuance. In two cases of semile gangrene it was signally useful as a disinfectant of rooms occupied by patients. But, far more with the well than the sick are the good effects of ozone observable. That it is the most powerful destroyer of the volatile elements of contagion known to our art I have no doubt. I have never known a second case of typhoid fever to occur in a house when one was treated in ozonized air unless the period of apparent incubation was reached before the first case was treated; nor the second case of diphtheria. Of scarlet fever I cannot speak so confidently, and yet, if I can obtain a fair isolation of the first child attacked, I expect to limit the cases to that. But I can say with great confidence that with hardly an exception cases subsequent to the first are greatly diminished in severity.

I have seen a case of malignant scarlet fever, fatal in thirty-six hours, the child rolling in convulsions, in its liquid excrements, succeeded in forty-eight hours by a second case of same nature, but perfectly amenable to treatment, though the disease was prolonged for weeks; and a third case in same family so slightly ill as to call for no treatment, and the fourth and youngest escape altogether. We may be prepared for such statements when we read that recent experiments show that the bacteria from the malignant pustule itself may be rendered harmless when propagated for a few hours in pure and free air.

I quote from a paper read May 10, 1883, before the Polytechnic Association of the American Institute, by William C. Conant:

"The most wonderful achievement of recent investigation reveals a philosophy of both bane and antidote that astonishes us with its simplicity as much as with its efficiency. At the moment when humanity stands aghast at the announcement that germs are not destroyed by disinfectants, comes the counter discovery that they are rendered harmless by oxygen. It seems that it makes no difference, really, of what sort or from what source are the bacteria that we take into the blood. The only material difference to us depends on the sort of atmosphere in which their hourly generations are bred. For example, the bacteria developed in confined air, from a simple infusion of hay, are found by experiment to be as capable of generating that most terrible of blood poisoners, the malignant pustule, as are the bacteria taken from the pustule itself.

"On the other hand, the bacteria from the malignant pustule itself, after propagating for a few hours in pure and free air, become a perfectly harmless race, and are actually injected into the blood with impunity. The explanation of the strange discovery is this—note its extreme simplicity—bacteria bred in copious oxygen perish for want of it as soon as they enter the blood vessels; whereas those inured to an unventilated atmosphere for a few generations, which means only a few hours, are prepared to thrive and propagate infinitely within our veins; and that is the whole mystery of blood poisoning and zymotic diseases."

When we consider that these minute organisms are stated by eminent microscopists to swarm in the blood by millions, estimated by Mr. Dancer at 3,700,000 in the breath of a human being in a single hour, over 60,000 a minute and 2,000 in a a single breath, propagating in the blood and consuming its oxygen, we may well doubt the efficacy of any antidote, internally administered, which has proved externally to the body to have no effect. We are drawn irresistibly to the study of the treatment of disease by modified atmospheres, not necessarily ozone, but volatilized, respirable or irrespirable agents.

The next decade will witness a marvelous advance in this splendid field of experiment, and by and by we shall wonder at our ignorance and blindness in confining, practically, all our efforts in the cure of disease to remedies administered by the stomach. As surely as to-morrow comes, this shall come.

Thanks to the discoveries of Pasteur, Tyndall, Koch, and the long list of noble men who, though not of our profession, have incalculably benefited it by their researches, we shall ultimately cure all zymotic diseases.

While ozone may not be justly claimed a direct reinforcement to human vitality, it indirectly aids it by diminishing the influence of zymotic poisons. While it is doubtless true that concentrated ozone will destroy life in the mammalia, I would reason thus: it may be so employed in disease as to be harmless, and yet destructive to minute microscopic disease germs. Again, I argue that its admitted oxidizing power, by modifying an atmosphere favorable to *generating* germs, may largely or entirely prevent further propagation, or so modify the germ that it may be innocuous. All experiments prove that zymotic diseases lose their virulence as we rise to higher altitudes until, as Tyndall's experiments show, we may expose the ordinary test infusions without the possibility of fertilization.

To what is this due? certainly not to *temperature* alone. Ozone is most abundant in the upper atmosphere, and by its specific gravity is constantly descending to combine with oxidable gases and materials. I find by experiment that I can remove all foul odors in a cellar by locating a generator in the second or third story of a building. I only reiterate the opinion of Fox and all specialists in the study of ozone, that it should be diffused in all crowded and unwholesome hospitals, schools, prisons, mines; in all places where ordinary atmosphere is unequal to needed purification. I believe it to be entirely practical to ozonize the outer atmosphere of a plague-smitten city, and proposed in the recent yellow fever epidemics to do this in New Orleans and Memphis, but my ideas were deemed utopian. I patiently wait for time to do justice to the correctness of my opinions. I expect to show that not only in zymotic, but in what we term constitutional disease, ozone will surpass all present remedial agents; not only by direct action through the respiration, but by its wonderful influence upon remedial agents circulating in the blood. Ozone attacks metallic mercury with great promptness. May it not do the same while it, or its oxides, is within the body? I know it will oxidize what is known as amalgam, or soft filling, in dental cavities. It is, temporarily at least, a powerful aphrodisiac, which would indicate a stimulating effect upon the nervous system. I believe in disease it lessens the pulse and lowers vital temperature, but this I cannot assert. Many of my experiments have been made under such unfavorable environments—for instance, in filthy abodes where the ozone developed was so rapidly absorbed by previously existing putrescence, that it was exceedingly difficult to measure its value; but in experiments I intend soon to make in insulated receptacles, I hope to give more exact results. We must be patient—the mysteries which environ the study of ozone are immense.

Six years ago, at the suggestion of the late Dr. White, of this city, a committee was appointed, by the American Medical Association, to study and report upon ozone. Unless something definite was reported at the last meeting, nothing has been accomplished. Obtain, if it is possible, undoubted tests of the degree of ozonization in the atmosphere at all times, of what practical utility will it be in the treatment of disease? "Canst thou bind the sweet influences of Pleiades, or loose the bands of Orion?" Can you train this splendid corsair of the atmosphere, and make it subservient to your will? Can you really credit it with sufficient power, as it exists normally, to specially modify an existing disease? Has it not been tolerated, through all the ages, without exerting anything more than a purifying, oxidizing influence; perfectly compatible with the existence of localized putrescence and impurity?

And yet, there is confident expectation of something to be revealed. "The air is sweet with prophecies." The great army of explorers knows no rest. Day by day, it advances its lines, it establishes its posts, its camp fires gleam in the distance. Personally, I have no doubt, that the deadly fever of the tropics, the stealthy typhoid of civilization, the malarias of the swamps, the terror of maternity, puerperal fever, scarlatina, diphtheria, variola, even the dreaded cholera may be controlled or modified by oxidizing atmospheres; nor should we deem anything impossible in these days of illimitable progress. We "paint with the sunbeam," hold converse by the lightning's flash, and leap with the jubilant spirit of the waters from zone to zone. We know the anatomy of the microscopic inhabitants of the ocean's floor, and the spectral splendors of the sun. Deep under the dividing tides, on the great sympathetic nerves of the world, quiver and pass the thoughts, the aspirations, the events of all the continents. We shall persevere! We shall battle! We shall triumph! till,

> "Standing on what too long we bore,
> With shoulders bent and downcast eyes,
> We may perceive, unseen before,
> The path to higher mysteries."

